# Trajectory of NAFLD characteristics after Roux-en-Y gastric bypass: a five-year historical cohort study

**DOI:** 10.1590/1516-3180.2021.0828.07012022

**Published:** 2022-09-12

**Authors:** Fernanda Kreve, Guilherme Hoverter Callejas, Laísa Simakawa Jimenez, Rodolfo Araújo Marques, Felipe David Mendonça Chaim, Murillo Pimentel Utrini, Martinho Antonio Gestic, Almino Cardoso Ramos, Elinton Adami Chaim, Everton Cazzo

**Affiliations:** I MD. Resident Physician, Department of Surgery, Faculty of Medical Sciences, Universidade Estadual de Campinas (UNICAMP), Campinas (SP), Brazil.; II MD. Postgraduate Student, Department of Surgery, Faculty of Medical Sciences, Universidade Federal de Campinas (UNICAMP), Campinas (SP), Brazil.; III MD, MSc. Postgraduate Student, Department of Surgery, Faculty of Medical Sciences, State University of Campinas (UNICAMP), Campinas (SP), Brazil.; IV BM. Undergraduate Student, Department of Surgery, Faculty of Medical Sciences, Universidade Estadual de Campinas (UNICAMP), Campinas (SP), Brazil.; V MD, PhD. Attending Physician, Department of Surgery, Faculty of Medical Sciences, Universidade Estadual de Campinas (UNICAMP), Campinas (SP), Brazil.; VI MD. Attending Physician, Department of Surgery, Faculty of Medical Sciences, Universidade Estadual de Campinas (UNICAMP), Campinas (SP), Brazil.; VII MD, MSc. Attending Physician, Department of Surgery, Faculty of Medical Sciences, Universidade Estadual de Campinas (UNICAMP), Campinas (SP), Brazil.; VIII MD, PhD. Visiting Professor, Department of Surgery, Faculty of Medical Sciences, Universidade Estadual de Campinas (UNICAMP), Campinas (SP), Brazil.; IX MD, PhD. Full Professor, Department of Surgery, Faculty of Medical Sciences, Universidade Estadual de Campinas (UNICAMP), Campinas (SP), Brazil.; X MD, PhD. Adjunct Professor, Department of Surgery, Faculty of Medical Sciences, Universidade Estadual de Campinas (UNICAMP), Campinas (SP), Brazil.

**Keywords:** Gastric bypass, Bariatric surgery, Obesity, Fatty liver, Non-alcoholic fatty hepatopathy, Roux-en-Y gastric bypass, Hepatic steatosis

## Abstract

**BACKGROUND:**

The long-term effects of bariatric surgery on the course of non-alcoholic fatty hepatopathy (NAFLD) are not fully understood.

**OBJECTIVE:**

To analyze the evolution of NAFLD characteristics through noninvasive markers after Roux-en-Y gastric bypass (RYGB) over a five-year period.

**DESIGN AND SETTING:**

Historical cohort study; tertiary-level university hospital.

**METHODS:**

The evolution of NAFLD-related characteristics was evaluated among 49 individuals who underwent RYGB, with a five-year follow-up. Steatosis was evaluated through the hepatic steatosis index (HSI), steatohepatitis through the clinical score for non-alcoholic steatohepatitis (C-NASH) and fibrosis through the NAFLD fibrosis score (NFS).

**RESULTS:**

91.8% of the individuals were female. The mean age was 38.3 ± 10 years and average body mass index (BMI), 37.4 ± 2.3 kg/m^2^. HSI significantly decreased from 47.15 ± 4.27 to 36.03 ± 3.72 at 12 months (P < 0.01), without other significant changes up to 60 months. C-NASH significantly decreased from 0.75 ± 1.25 to 0.29 ± 0.7 at 12 months (P < 0.01), without other significant changes up to 60 months. NFS decreased from 1.14 ± 1.23 to 0.27 ± 0.99 at 12 months (P < 0.01), and then followed a slightly ascending course, with a marked increase by 60 months (0.82 ± 0.89), but still lower than at baseline (P < 0.05). HSI variation strongly correlated with the five-year percentage total weight loss (R = 0.8; P < 0.0001).

**CONCLUSION:**

RYGB led to significant improvement of steatosis, steatohepatitis and fibrosis after five years. Fibrosis was the most refractory abnormality, with a slightly ascending trend after two years. Steatosis improvement directly correlated with weight loss.

## INTRODUCTION

Non-alcoholic fatty liver disease (NAFLD) is the most common liver disease worldwide and is expected to be the most frequent indication for liver transplantation by 2030. NAFLD is closely related to obesity, metabolic syndrome and type 2 diabetes mellitus (T2DM). In this setting, its prevalence ranges from about 50% to up to 90%, which has made NAFLD a challenging public health concern, as the prevalence of obesity and overweight has increased to epidemic levels over the last few decades.^1,2^

NAFLD encompasses a spectrum of histopathological abnormalities ranging from simple steatosis to cirrhosis, end-stage liver failure and even liver cancer. Its diagnosis is based on the presence of abnormal fat accumulation in more than 5% of hepatocytes on liver biopsy, excluding other causes such as alcohol abuse, viral infection, autoimmune disease or drug-induced liver disease.^3^ Although liver biopsy is the gold standard for diagnosing NAFLD, it is an invasive test and not free from complications.

In this context, noninvasive markers have been developed. These are mostly calculated through routine laboratory tests and clinical and anthropometric assessments. They help to identify patients at higher risk of presenting the severe forms of the disease, and to monitor disease progression.^4^

Bariatric surgery is an effective treatment for severe and refractory obesity and leads to sustained weight loss with potential reductions in liver fat, inflammation and fibrosis. Significant evidence of the beneficial effects of Roux-en-Y gastric bypass (RYGB) on NAFLD has been demonstrated through studies with follow-ups of up to two years, but evidence based on lengthier follow-up times is scarcer. Considering that weight regain, obesity recidivism and re-emergence of insulin resistance and T2DM may occur after this two-year “honeymoon” period, a longer-term analysis of the effect of surgery on the trajectory of aspects of the NAFLD spectrum is of great importance.^5,6,7^

## OBJECTIVE

The aim of this study was to analyze the evolution of different NAFLD characteristics through noninvasive markers among patients who underwent Roux-en-Y gastric bypass, over a five-year period.

## METHODS

### Study design

This was an observational historical cohort study based on a prospectively collected database. It evaluated the clinical and laboratory characteristics of individuals who underwent open RYGB, with a five-year follow-up, at a tertiary-level university hospital from July 2014 to March 2015. The research project was evaluated and approved by our institutional ethics review board on September 24, 2020 (CAAE: 37900820.8.0000.540).

Comparisons were made between the period immediately before surgery and the times of 12, 24, 36, 48 and 60 months afterwards, to measure the impact of the procedure on NAFLD, through the evolution of noninvasive markers. An analysis of the diagnostic accuracy of each score at the time of surgery was carried out using liver biopsies collected systematically during surgery.

### Study population

Individuals of both genders, aged between 18 and 70 years, who had undergone RYGB were included. Patients with the following characteristics were excluded: a history of alcohol or hepatotoxic drug use; chronic viral hepatitis or serological abnormalities; a diagnosis of current or past biliary obstruction; belonging to vulnerable groups (mental patients, institutionalized or under 18 years old); incomplete medical records; and follow-up of less than five years. Out of the 90 individuals who underwent a bypass within the period considered, 49 fulfilled the criteria and were assessed in this study.

All patients who undergo bariatric surgery in our institution take part in a preoperative weight loss program that lasts from 4 to 12 weeks, consisting of weekly consultations carried out by a multidisciplinary team. Individuals undergo surgery when they reach a minimum preoperative weight loss of 10%, and provided that they have a minimum body mass index (BMI) of 35 kg/m^2^ with obesity-related morbidities, or 40 kg/m^2^. All procedures were performed by the same surgical team and followed the same technique.

### Liver biopsy

A wedge liver biopsy is performed during surgery immediately after the main procedure. A fragment of length 2 cm is extracted using blunt scissors, usually from segment III or IV of the liver, and hemostasis is subsequently performed.

### Variables

#### Histopathological analysis

Changes relating to NAFLD were classified into categories, according to the classification system of the Brazilian Society of Hepatology: 1) steatosis (absent, mild, moderate or intense); 2) fibrosis (according to the Kleiner-Brunt classification: 0 - absent; 1 - isolated perisinusoidal or periportal; 2 - periportal and perisinusoidal; 3 - presence of fibrous septa (“bridging fibrosis”); or 4 - cirrhosis); and 3) steatohepatitis (classified in degrees: 0, 1+, 2+ or 3+). Kleiner-Brunt grades 3 and 4 are considered to represent advanced fibrosis.^8,9^

#### Noninvasive markers

The results observed from liver biopsies were correlated with the results obtained using the noninvasive markers, i.e. the hepatic steatosis index (HSI), NAFLD fibrosis score (NFS) and clinical score for non-alcoholic steatohepatitis (C-NASH), at the baseline. Each marker was calculated throughout the five-year follow-up: at baseline, 12, 24, 36, 48 and 60 months. Table 1 shows how each of them was calculated, its rationale and the respective cutoff values adopted.

**Table 1.** Main characteristics of each noninvasive score assessed

Score	Rationale	Calculation method		Cutoff values and interpretation
**HSI**	Designed by Lee et al.^28^ in 2010 to predict occurrence of steatosis in the general population.	HSI = 8 * ALT/AST + BMI (+ 2 if T2DM and + 2 if female)		A score > 36 indicates the presence of steatosis, while a score < 30 indicates absence of steatosis
**NFS**	Developed by Angulo et al.^29^ in 2007 to predict advanced fibrosis in NAFLD patients.	NFS = -1.675 + 0.037 * age (years) + 0.094 * BMI (kg/m^2^) * IGT/T2DM (yes = 1 or no = 0) + 0.99 * AST/ALT – 0.013 * platelet count (* 10^9^/l) – 0.66 * albumin (g/dl)		A score > 0.676 indicates advanced fibrosis, while a score < -1.455 excludes advanced fibrosis
**C-NASH**	Created by Tai et al.^30^ in 2017 to predict occurrence of NASH based on clinical characteristics.	Clinical aspect	Points	A sum of points ≥ 3 indicates the presence of steatohepatitis
		BMI (kg/m^2^) 40–45 > 45 AST > 40 IU/l Triglycerides > 140 mg/dl	1 2 2 1	

* = multiplication sign; NAFLD = non-alcoholic fatty liver disease; NASH = non-alcoholic fatty liver steatohepatitis; HSI = hepatic steatosis index; NFS = non-alcoholic fatty liver disease fibrosis score; C-NASH = clinical score for non-alcoholic steatohepatitis; BMI = body mass index; ALT = alanine aminotransferase; AST = aspartate aminotransferase; T2DM = type 2 diabetes mellitus; IGT = impaired glucose tolerance.

#### Anthropometric and biochemical characteristics

The anthropometric characteristics evaluated were weight, BMI and percentage total weight loss (%TWL). The laboratory tests evaluated included fasting glucose (FG), aspartate aminotransferase (AST), alanine aminotransferase (ALT), triglycerides, serum albumin and platelet count.

#### Statistical analysis

Descriptive analysis was performed, with presentation of frequency tables for categorical variables and position and dispersion measurements for numerical variables. To compare proportions, the chi-square test was used, or Fisher’s exact test when necessary. To compare continuous measurements between two evaluation times, the Mann-Whitney test was used. For comparison between three or more evaluation times, the Kruskal-Wallis test was used, with Tukey’s post-test analysis when significant. For analysis of correlations between continuous variables, linear regression models were used. To assess the reliability of each score at baseline, diagnostic accuracy measurements (sensitivity, specificity, positive and negative predictive values and overall accuracy) were calculated considering histopathological examination as the gold-standard diagnostic method. The significance level adopted for the statistical tests was 5% (P < 0.05). The SAS System for Windows software (Statistical Analysis System), version 9.2, was used (SAS Institute Inc., 2002-2008; Cary, North Carolina, United States).

## RESULTS

Out of the 49 patients selected for this study, 45 (91.8%) were female. The patients’ mean age was 38.3 ± 10 years, and their BMI was 37.4 ± 2.3 kg/m^2^. Regarding comorbidities, 28 (57.1%) had hypertension, 13 (26.5%) presented T2DM and 29 (59.2%) had some form of dyslipidemia. In the histopathological analysis at the time of surgery, 41 patients (83.7%) presented steatosis and 23 (46.9%) had steatohepatitis. Fibrosis was present in 33 patients (67.3%).

**Figure 1.**
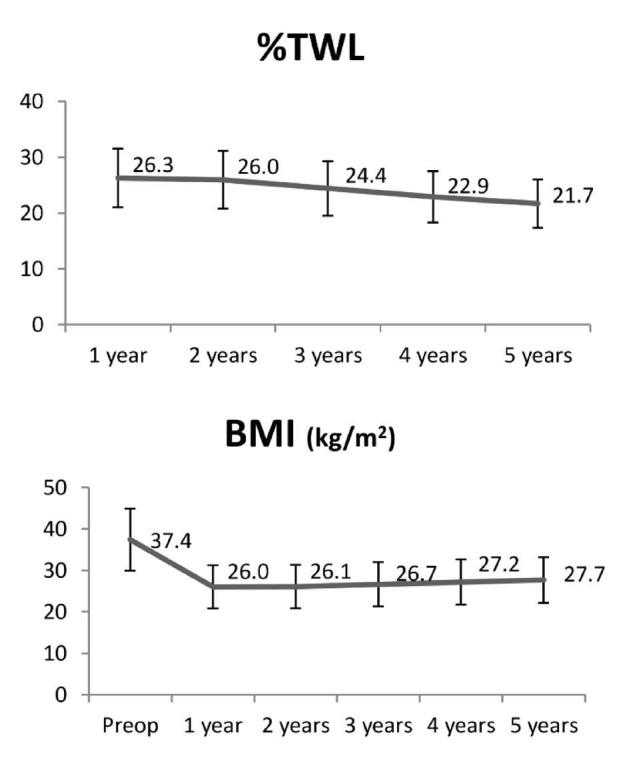
Evolution of percentage total weight loss (%TWL) and body mass index (BMI) over time.

With regard to weight loss during follow-up, the minimum weight was achieved after one to two years, followed by a slight increase up to five years, which was not statistically significant (Table 2). Figure 1 presents a graphical representation of the %TWL over the course of the follow-up.

**Table 2.** Evolution of body mass index, percentage of total weight loss, prevalence of non-alcoholic fatty hepatopathy (NAFLD) features and values of NAFLD markers over the five-year follow-up

	Baseline	1 year	2 years	3 years	4 years	5 years	P value
BMI (kg/m^2^)	37.4 ± 2.3	26 ± 2.7	26.1 ± 2.9	26.7 ± 2.7	27.2 ± 2.8	27.7 ± 3.2	< 0.0001 (BL vs. 1 y: P < 0.01; BL vs. 2 y: P < 0.01; BL vs. 3 y: P < 0.01; BL vs. 4 y: P < 0.01; BL vs. 5 y: P < 0.01)
%TWL (%)	Not applicable	26.3 ± 8.3	26 ± 8.7	24.4 ± 8.1	22.9 ± 8.9	21.7 ± 9.7	< 0.0001 (1 y vs. 4 y: P < 0.01; 1 y vs. 4 y: P < 0.01; 2 y vs. 4 y: P < 0.01; 2 y vs. 5 y: P < 0.01; 3 y vs. 5 y: P < 0.01)
HSI	47.14 ± 4.27	36.03 ± 3.72	35.9 ± 4.29	35.63 ± 3.98	35.86 ± 3.6	36.3 ± 4.38	< 0.0001 (BL vs. 1 y: P < 0.01; BL vs. 2 y: P < 0.01; BL vs. 3 y: P < 0.01; BL vs. 4 y: P < 0.01; BL vs. 5 y: P < 0.01)
C-NASH	0.76 ± 1.25	0.29 ± 0.71	0.12 ± 0.48	0.06 ± 0.32	0.06 ± 0.32	0.14 ± 0.46	< 0.0001 (BL vs. 1 y: P < 0.01; BL vs. 2 y: P < 0.01; BL vs. 3 y: P < 0.01; BL vs. 4 y: P < 0.01; BL vs. 5 y: P < 0.01)
NFS	1.137 ± 1.228	0.269 ± 0.996	0.476 ± 1.043	0.640 ± 1.031	0.786 ± 1.016	0.821 ± 0.889	< 0.0001 (BL vs. 1 y: P < 0.01; BL vs. 2 y: P < 0.01; BL vs. 3 y: P < 0.01; BL vs. 4 y: P < 0.05; BL vs. 5 y: P < 0.05; 1 y vs. 3 y: P < 0.05; 1 y vs. 4 y: P < 0.01; 1 y vs. 5 y: P < 0.01; 2 y vs. 5 y: P < 0.05)
Steatosis according to HSI – n (%)	49 (100%)	21 (42.9%)	20 (40.8%)	20 (40.8%)	19 (38.8%)	19 (38.8%)	< 0.0001 (BL vs. 1 y: P < 0.01; BL vs. 2 y: P < 0.01; BL vs. 3 y: P < 0.01; BL vs. 4 y: P < 0.01; BL vs. 5 y: P < 0.01)
Steatohepatitis according to C-NASH – n (%)	10 (20.4%)	0	0	0	0	0	< 0.0001 (BL vs. 1 y: P < 0.01; BL vs. 2 y: P < 0.01; BL vs. 3 y: P < 0.01; BL vs. 4 y: P < 0.01; BL vs. 5 y: P < 0.01)
Fibrosis according to NFS – n (%)	33 (67.3%)	16 (32.7%)	16 (32.7%)	24 (49%)	26 (53.1%)	29 (59.2%)	0.001 (BL vs. 1 y: P < 0.01 BL vs. 2 y: P < 0.01)

BMI = body mass index; %TWL = percentage total weight loss; NAFLD = non-alcoholic fatty liver disease; NASH = non-alcoholic fatty liver steatohepatitis; HSI = hepatic steatosis index; NFS = non-alcoholic fatty liver disease fibrosis score; C-NASH = clinical score for non-alcoholic steatohepatitis; vs. = versus; N = number of individuals; BL = baseline; y = year. Preop = preoperative.

Preop = preoperative.

Analysis on the diagnostic accuracy of the tests in comparison with surgical biopsy showed that the overall accuracies of the tests were as follows: 83.7% for HSI to detect steatosis, 67.7% for NFS to detect advanced fibrosis and 73.5% for C-NASH to detect steatohepatitis. In Table 3, the detailed results from this analysis are presented.

**Table 3.** Diagnostic accuracy of noninvasive markers for aspects of non-alcoholic fatty liver disease

		Value	95% confidence interval
**HSI**	Sensitivity	100%	91.4%–100%
	Specificity	0	0%–36.9%
	Positive likelihood ratio	1	Not applicable
	Negative likelihood ratio	Not applicable	Not applicable
	Positive predictive value	83.7%	83.7%–83.7%
	Negative predictive value	Not applicable	Not applicable
	Overall accuracy	83.7%	70.3%–92.7%
**C-NASH**	Sensitivity	43.5%	23.2%–65.5%
	Specificity	100%	86.8%–100%
	Positive likelihood ratio	Not applicable	Not applicable
	Negative likelihood ratio	0.6	0.4–0.8
	Positive predictive value	100%	Not applicable
	Negative predictive value	66.7%	58.3%–74.1%
	Overall accuracy	73.5%	58.9%–85.1%
**NFS**	Sensitivity	100%	84.6%–100%
	Specificity	8.3%	0.2%–38.5%
	Positive likelihood ratio	1.1	0.9–1.3
	Negative likelihood ratio	0	Not applicable
	Positive predictive value	66.7%	62.8%–70.3%
	Negative predictive value	100%	Not applicable
	Overall accuracy	67.7%	49.5%–82.6%

HSI = hepatic steatosis index; NFS = non-alcoholic fatty liver disease fibrosis score; C-NASH = clinical score for non-alcoholic steatohepatitis. Preop = preoperative.

**Figure 2.**
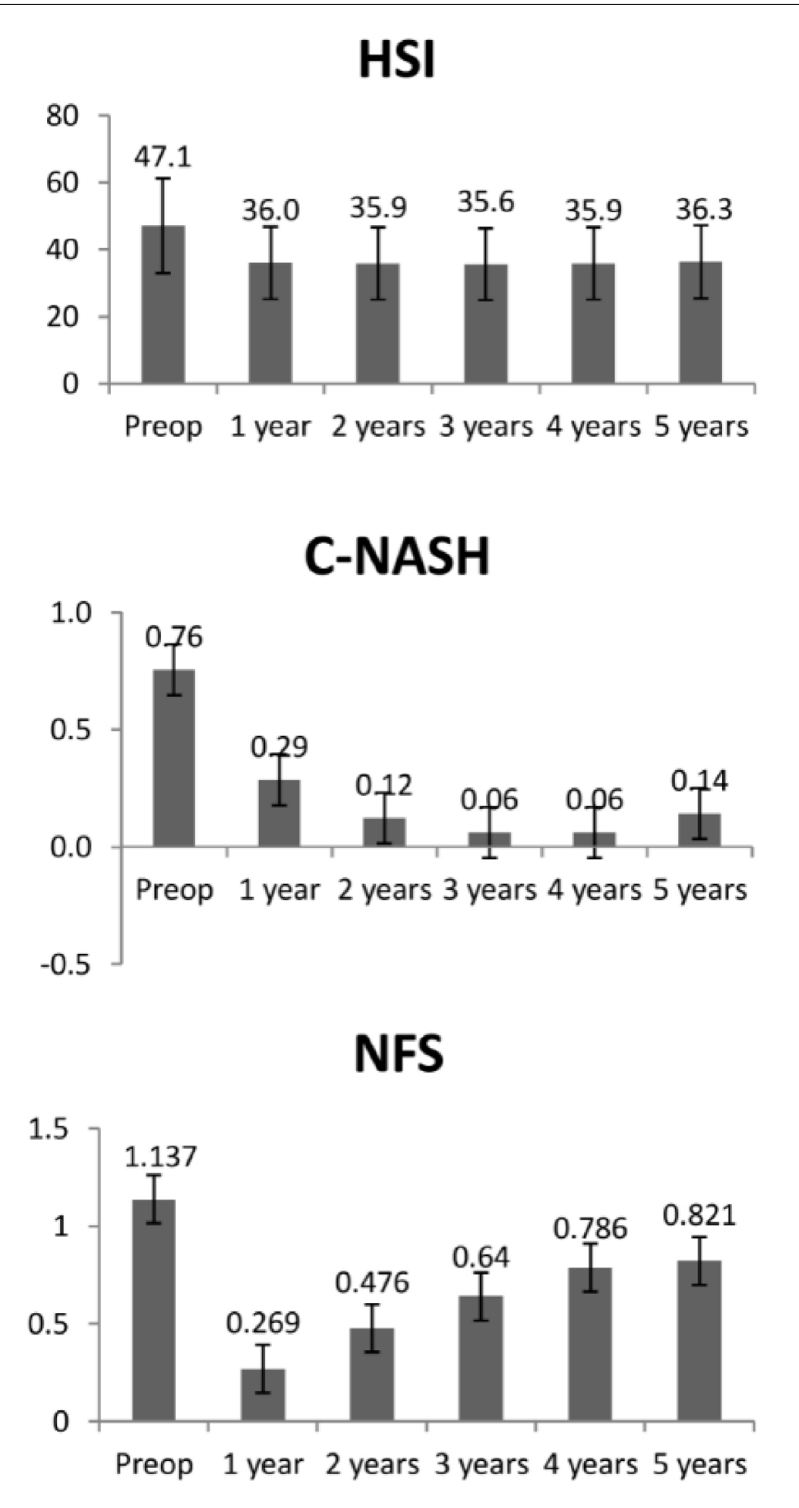
Evolution of hepatic steatosis index (HSI), clinical score for non-alcoholic steatohepatitis (C-NASH) and non-alcoholic fatty liver disease fibrosis score (NFS) over time.

There was a marked and statistically significant reduction in noninvasive NAFLD scores in the first year, such that HSI decreased from 47.15 ± 4.27 preoperatively to 36.03 ± 3.72 at 12 months (P < 0.01), without any further significant change from then until 60 months. C-NASH significantly decreased from 0.75 ± 1.25 preoperatively to 0.29 ± 0.7 at 12 months (P < 0.01); thereafter, there were no further significant changes in its values up to 60 months. NFS decreased from 1.14 ± 1.23 preoperatively to 0.27 ± 0.99 at 12 months (P < 0.01); from then onwards, it entered a slightly ascending trajectory, yielding a marked increase at 60 months (0.82 ± 0.89), which was significantly higher than the levels observed at 12 months (P < 0.01) and 24 months (P < 0.05), but still significantly lower than what had been seen preoperatively (P < 0.05). In Figure 2, graphical representations of the evolution of the three markers over the follow-up are presented. Table 2 presents the detailed evolution of each marker over the follow-up, along with the prevalence of each aspect of NAFLD, according to the cutoff values of the noninvasive markers.

Preop = preoperative.

With regard to possible correlations between the variation in NAFLD scores and weight loss over the total follow-up period, there was a strong positive correlation between the variation in the HSI and the five-year %TWL (R = 0.8; P < 0.0001). On the other hand, there were no significant correlations between the five-year %TWL and variations in C-NASH (R = 0.1; P = 0.7) and NFS (R = 0.1; P = 0.7).

## DISCUSSION

NAFLD, one of the most common causes of chronic liver disease, leads to increased long-term morbidity and mortality. The current evidence suggests that bariatric surgery reduces the degree of fat deposition, liver inflammation and fibrosis, in agreement with the findings from the present study, which revealed a marked reduction in NAFLD marker scores in the first two years after surgery.^10^ With regard to weight loss, the findings were also comparable to the literature, demonstrating maximum weight loss between one and two years postoperatively, followed by mild to moderate weight recovery. Despite this recovery, there was no significant difference in mean BMI and %TWL between the second and fifth years of follow-up, which emphasizes that the satisfactory results obtained through RYGB were maintained over this follow-up period.^11,12^

Uehara et al. conducted a prospective study with a five-year follow-up among 102 patients in Japan in which the evolution of NAFLD was analyzed through liver enzymes. They demonstrated that the reduction in enzymes was sustained over the follow-up, with a slight tendency to increase seen at five years.^13^ In a prospective cohort study, Mathurin et al. evaluated 211 patients who underwent various bariatric surgery techniques and who had a paired biopsy available from a fifth-year follow-up. They found improvement in steatosis, ballooning and inflammation, with a significant reduction in the percentage of patients with NASH, compared with the preoperative assessment.^14^

One important finding from the present study was a strong correlation between %TWL and the variation in the HSI, such that the greater the weight loss was, the greater the drop in this score also was. The absence of this same correlation between %TWL and the variation in the other scores (C-NASH and NFS) seems to relate to their dependence on factors other than weight loss. Histological studies would be more indicated for confirming these findings, but they are invasive and with a potential risk of complications.

This directly proportional relationship between steatosis and weight trajectory was also demonstrated by Yoshioka et al., in a five-year cohort study that observed improvement or worsening of NAFLD over time after weight loss or gain exclusively through changes of lifestyle, respectively.^15^ Van-Wagner et al., in a large cohort study that followed 4,423 individuals over 25 years, had also suggested that the trajectory of BMI over the course of life was a reliable predictor of the risk of development and worsening of hepatic steatosis, especially when an ascending path was manifested at an early age.^16^ Bariatric surgery studies based on paired biopsies during and after the procedure showed results comparable to those of the current study.^17^ The improvement in steatohepatitis, although not proportional to the volume of weight loss, was also significant and was maintained over the follow-up. Lassailly et al. previously demonstrated in a paired biopsy study that improvement in steatohepatitis was consistent over a five-year follow-up in a group of 64 patients undergoing various procedures.^18^

The current study also showed that fibrosis is the most refractory abnormality among the long-term effects of bariatric surgery. While steatosis and steatohepatitis markers show an initial drop that remains constant, the NFS score shows a sharp drop in the first postoperative year, followed by subsequent slightly progressive increases in its values; nonetheless, at the end of the follow-up, the NFS values are still significantly better than at the baseline. A study carried out in 2019 by Yeo et al. demonstrated similar short-term results, showing that the correlation between weight loss and significant improvement in the NFS score only occurred in the first postoperative year.^19^ Fakhry et al., in a meta-analysis of prospective studies that enrolled 2,374 patients, observed virtually universal findings pointing to almost complete reversal or improvement of steatosis and steatohepatitis after bariatric surgery, while fibrosis progressed with improvement or reversal in only 30% of the individuals.^20^

A previous study by our group revealed comparable results over a short-term one-year follow-up, with fibrosis reversal in 55%; an expansion of this same study with a three-year follow-up showed that weight regain was associated with worse results, although the benefits in relation to the baseline were still clear.^21,22^ Considering the variables that make up the NFS calculation (age, BMI, glucose intolerance and/or diabetes, liver enzymes, platelet count and albumin), it can be postulated that individuals’ aging and possible recovery of weight after 24 months, and the possible re-emergence of glucose metabolism disorders after this period play significant roles in mitigation of previous beneficial effects. However, it should be considered that, despite this not so marked improvement, the surgery still proved to be beneficial, since the mean score remained significantly lower than that observed in the preoperative period.

Despite this relative refractoriness of liver fibrosis to the effects of the surgical procedure, largely due to its inherently poorly reversible scarring characteristic, it is possible to speculate that, even in these cases where the outcomes were not so favorable, there was at least a tendency towards stability of this abnormality. Sanyal et al. demonstrated in a landmark study that about 20% of individuals with NAFLD-related F3 liver fibrosis progress to cirrhosis over 96 months if the disease follows its natural path.^23^ Given this natural history of fibrosis, the simple absence of progression of the same degree of fibrosis should be considered to be a significant benefit for this group of individuals.

As shown in systematic reviews over the years, the effects of bariatric surgery and weight loss on NAFLD improvement are significant, thus constituting a factor that directly impacts the long-term survival of these patients.^24,25^ Use of noninvasive markers has become an important tool in the long-term follow-up of these patients, as it is an easily applicable and risk-free method that allows assessment of the evolution of several NAFLD-related features and helps in understanding the effects of the surgical procedure. Furthermore, in individuals with suggestive clinical and/or imaging findings, the scores can help to define the cases for which a liver biopsy will be necessary and the ideal timing for it to be carried out.^26^

The current study had some limitations that must be considered. The retrospective design used in this study usually reduces the quality of the data considered, even if they were prospectively collected. Loss to follow-up led to a relatively small sample. Use of noninvasive markers instead of histopathological examination is not the ideal method for evaluating NAFLD, but the costs, risks and ethical issues associated with performing serial biopsies make the methods used here more practical and appropriate.^27^ Hence, considering the length of follow-up adopted for our study and the availability of all tests and indexes in the entire study population at all the time points considered, the study design was adequate and was able to provide an accurate representation of NAFLD-related features over time after RYGB.

## CONCLUSION

RYGB led to significant improvement of steatosis, steatohepatitis and fibrosis after five years. Fibrosis was the most refractory abnormality and presented a slightly ascending trend after two years. The improvement of steatosis directly correlated with weight loss.
